# Can aerosols-generating dental, oral and maxillofacial, and orthopedic surgical procedures lead to disease transmission? An implication on the current COVID-19 pandemic

**DOI:** 10.3389/froh.2022.974644

**Published:** 2022-08-01

**Authors:** Essam Ahmed Al-Moraissi, Amanjot Kaur, Frank Günther, Andreas Neff, Nikolaos Christidis

**Affiliations:** ^1^Department of Oral and Maxillofacial Surgery, Faculty of Dentistry, Thamar University, Dhamar, Yemen; ^2^Oral and Maxillofacial Surgery, Department of Dentistry, All India Institute of Medical Sciences, Jodhpur, India; ^3^Medical Microbiology and Hygiene, Marburg University Hospital, Marburg, Germany; ^4^Department of Oral and Maxillofacial Surgery, University Hospital Marburg Universitätsklinikum Giessen und Marburg GmbH, Marburg, Germany; ^5^Division of Oral Diagnostics and Rehabilitation, Department of Dental Medicine, Karolinska Institutet, Huddinge, Sweden

**Keywords:** COVID-19, maxillofacial surgery, aerosols, aerosol generating dental procedure, systematic review, orthopedic, coronavirus disease 2019 (COVID-19), SARS-CoV-2

## Abstract

Various dental, maxillofacial, and orthopedic surgical procedures (DMOSP) have been known to produce bioaerosols, that can lead to the transmission of various infectious diseases. Hence, a systematic review (SR) aimed at generating evidence of aerosols generating DMOSP that can result in the transmission of severe acute respiratory syndrome coronavirus 2 (SARS-CoV-2), further investigating their infectivity and assessing the role of enhanced personal protective equipment (PPE) an essential to preventing the spreading of SARS-CoV-2 during aerosol-generating procedures (AGPs). This SR was performed according to the Preferred Reporting Items for Systematic Reviews and Meta-Analyses statement (PRISMA) guidelines based on a well-designed Population, Intervention, Comparison, Outcomes and Study (PICOS) framework, and various databases were searched to retrieve the studies which assessed potential aerosolization during DMOSP. This SR included 80 studies (59 dental and 21 orthopedic) with 7 SR, 47 humans, 5 cadaveric, 16 experimental, and 5 animal studies that confirmed the generation of small-sized < 5 μm particles in DMOSP. One study confirmed that HIV could be transmitted by aerosolized blood generated by an electric saw and bur. There is sufficient evidence that DMOSP generates an ample amount of bioaerosols, but the infectivity of these bioaerosols to transmit diseases like SARS-CoV-2 generates very weak evidence but still, this should be considered. Confirmation through isolation and culture of viable virus in the clinical environment should be pursued. An evidence provided by the current review was gathered by extrapolation from available experimental and empirical evidence not based on SARS-CoV-2. The results of the present review, therefore, should be interpreted with great caution.

## Introduction

Pandemics are a never-ending entity, as the viruses end up being part of the ecosystem. In less than two decades, the world has faced a SARS outbreak (2002), a MERS outbreak (2012) and finally, at present, the healthcare systems are struggling with severe acute respiratory syndrome coronavirus 2 (SARS-CoV-2). The oral mucous membrane has been reported to have a high affinity for angiotensin-converting enzyme receptor 2 (ACE2) which is responsible for the entrance of the virus into human cells, then starting its replications [1]. Thus, the saliva may contain more viral load, and oral and maxillofacial surgeons face a substantial risk of exposure to SARS-CoV-2, as their actual field of work is close to both the oral cavity and the nasopharynx/oropharynx [2].

Aerosol-generating procedures (AGPs) are defined as any medical, dental, or patient care procedure that yields in the generation of airborne particles ≤ 5 μm in size [3]. Particles < 5 μm are produced by several dental procedures, posing an increased risk of transmission of respiratory infections such as COVID-19 [4, 5]. Aerosols thus refer to liquid and solid particles ( ≤ 5 μm) which dehydrate and thus retain in the air for hours before falling on the ground in a larger distance (>>2 m or 6 feet, respectively) or entering the respiratory system, whereas, droplets are described as larger entities (>5 μm) that rapidly drop to the ground due to the force of gravity, typically 3–6 feet of the carrier [6]. Droplets and splatter are a mixture of air, water, saliva, and/or solid particles, becoming visible to the naked eye when >50 μm.

All dental surgical procedures performed with a high-speed rotating handpiece, ultrasonic scaler, and water air syringe are AGPs that are mostly contaminated with blood, bacteria, viruses, and fungi [7–12]. Similar bioaerosols are generated by various orthopedic procedures owing to the use of high-speed, power drilling and cutting tools, electrocautery, and pulse lavage [13–15]. It is already a known fact that bone and tooth cutting with high-speed burs in combination with external irrigation produces aerosols, further tossing the particles into space [16–20]. Thus, dental, maxillofacial, and orthopedic procedures (DMOSP) are at the highest risk with increased bacterial and viral load [21–24].

Literature evidence is supported by scattered data that DMOSP generates various amounts of bioaerosols [24–28]. The maxillofacial procedures include simple extractions to complex bone drilling and cutting procedures, and there is a scarcity of data for isolated oral and maxillofacial procedures. Hence, a single review compiling all data was the need of the hour.

Thus, a systematic review was conducted to identify whether there is scientific evidence supporting that DMOSP is AGPs and whether bioaerosols produced at DMOSP can transmit SARS-CoV-2, thus leading to the transmission of COVID-19. Furthermore, there is still a conspicuous lack of scientific evidence substantiating that enhanced PPE is necessary to protect during oral and maxillofacial surgery (OMFS) when dealing with suspected or confirmed patients with COVID-19 in this current pandemic outbreak. The authors hypothesize that enhanced PPE using respirators (N95 or FFP 2/3) would be sufficient and more effective than standard PPE without respirators or equivalents in protecting dentists and surgeons against SARS-CoV-2 during AGPs in suspected and confirmed COVID-19 cases.

## Methodology

**Figure 1:**
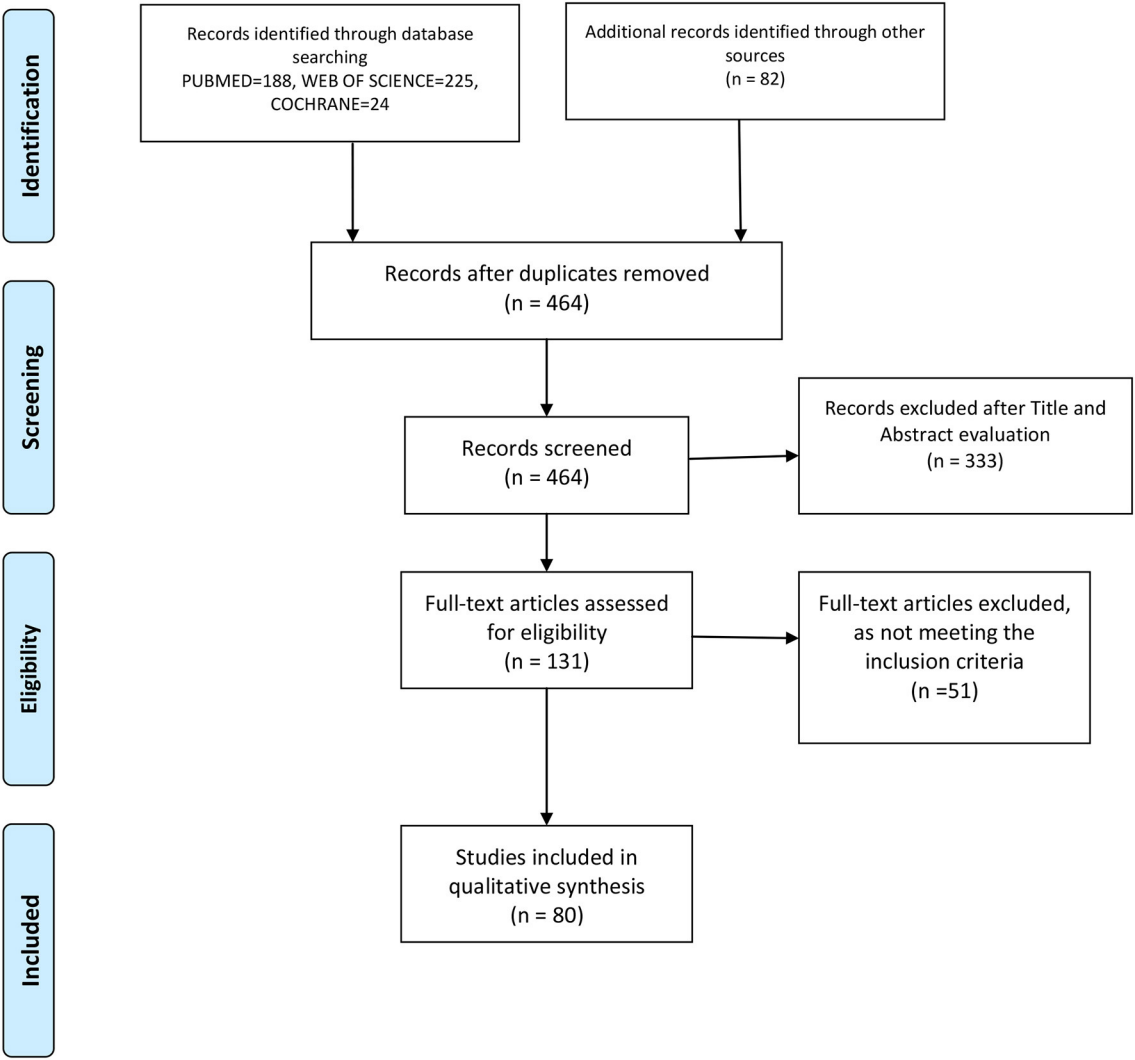
PRISMA flowchart of the included studies.

We performed this review following the latest Preferred Reporting Items for Systematic Reviews and Meta-Analyses statement (PRISMA) [29], and the flow diagram is shown in Figure 1, in combination with the Network Meta-Analyses of Health Care Interventions, and was registered in PROSPERO with No. CRD42020192912.

### Literature search

Two independent reviewers searched various databases, such as PUBMED, Cochrane, and Web of Science using the keywords “aerosols,” “bioaerosols,” “transmission,” “oral,” “microbes,” “maxillofacial,” and “orthopedics.” All the online databases of various oral and maxillofacial surgery journals (IJOMS, BJOMS, JOMS, and JOCMF) and orthopedic journals (Bone and Joint Journal, Spine, JBJS-America, and European Spine Journal) were searched robustly. All the gray literature was searched using references of the tentatively selected articles for further identification of potentially eligible articles, followed by random screening of the first 100 articles on Google Scholar. All English language articles whose full texts were available till 10th March 2022 were included in this study.

### Selection criteria

Based on the objective of this rapid systematic review, there were two inclusion criteria based on the research question (PICOS) as shown in Table 1.

**Table 1:** Focussed questions of the study and eligibility criteria.

Focused questions	1. Do DMOSP generate bioaerosols (and if so, which ones), which can result in transmission of SARS-CoV-2? 2. Are aerosolized airborne droplets (and to which extent is splatter) in DMOSP infective? 3. Is additional standard PPE an essential to prevent spreading of SARS-CoV-2 and thus COVID-19 during aerosol generating DMOSP?
Eligibility criteria	PICOS: Population (P): adult dental health care workers (HCW, defined as workers in a health care setting that could be exposed to patients with acute respiratory illness), such as oral and maxillofacial surgeons, dentists or dental assistants who are performing AGPs for unknown, negative, suspected or positive COVID-19 patients. Intervention (I): enhanced PPE include respirators such as N95 [certified by the National Institute for Occupational Safety and Health (NIOSH)], FFP2/FFP3 or powered air purifying respirator (PAPR), glove, water proof long sleeved gown, full face shield, head cap and overall cover. Comparator (C): Standard PPE including surgical face mask (certified for use as a medical device). Outcomes (O): The primary outcome was effectiveness of PPE against SARS-CoV-Study design (S): all literature sources discussing effectiveness of PPE against SARS-CoV, SARS-CoV-2, MERS-CoV. Additionally, national and international societies' recommendations, guidelines on using PPE for dental health care workers during the COVID-19 pandemic outbreak were included. Inpatients and outpatients.
	PICOS: Population (P): patients who underwent dental and oral and maxillofacial or orthopedic osteotomies using high speed devices. Intervention (I): high speed devices Comparator (C): not applicable. Outcomes (O): detection of aerosols and splatter and count of bacteria. Study design (S): all clinical human, cadaver and *in vitro* studies were included.

No restrictions were placed on the design of the article, publication year, or author's country. However, studies of non-English origin, conference abstracts, protocols, and case reports or lack of full-text availability were excluded from the current study.

### Data extraction

The following information was extracted: authors, type of study, how outcomes were measured, type of surgical procedures, type of surgical instruments used, conclusion, evidence of aerosol generation, evidence of transmission risk, and type of microbial species.

Any disagreement regarding the eligibility or data extracted was discussed among themselves to finalize a decision.

### Synthesis of results

The results were presented narratively to answer the prior authors' clinical questions as stated in Table 1. Inter-rater reliability was confirmed using the Kappa coefficient.

## Results

Figure 1 is a flowchart on the process of article evaluation for inclusion in the present systematic review. Based on the literature search, out of 131 articles assessed for eligibility, only 80 were included in the qualitative analysis.

### Characteristics of included studies

Characteristics of the included studies in this review have been summarized in Supplementary Table 1 with individual characteristics in Table 2.

**Table 2:** Characteristics of studies included in the systematic review (*n* = 80).

**S. no**.	**Characteristic**	**Number of studies**
1.	Study design	
	• Human study	47
	• Animal study	5
	• Cadaveric study	5
	• Experimental (lab) study	16
	• Systematic reviews/narrative reviews	7
2.	Study procedures	
	• Dental	59
	• Orthopedic	21
3.	Setting in which aerosol generation was evaluated	
	• Crown preparation	6
	• Access opening/restoration/enamel cutting	19
	• Orthodontic debonding	4
	• Extractions/oral surgery procedures	8
	• Unspecified dental procedures	11
	• Arthoplasty	11
	• Spine surgery	4
	• Trauma	1
	• Tenotomy	2
	• Unspecified orthopedic surgery	6
4.	Specific instrument evaluated for aerosols production	
	• Ultrasonic scalers	26
	• Micromotor	21
	• High speed air turbines	15
	• Triplex syringe	2
	• Orthodontic debonding pliers	2
	• Dental-not specified	4
	• Burr/cutter/ultrasonic devices for bone	3
	• SAW	5
	• Drill	9
	• Electrocautery	5
	• Irrigators	2

Systematic reviews are not calculated in this table.

### Characteristics of aerosols generated in DMOSP

#### Aerosols spread

Aerosol spread was evaluated by the majority of the studies, with most studies measuring spread of bacteria during surgery with Petri dishes placed at regular intervals to identify aerial spread of microbes [16–18, 20, 30–67], The other methods used to confirm aerosol spread were laser beam for illumination with photographic analysis [21, 68], shadowgraphy [19], sodium fluorescein for illumination [4, 69], UV illumnination [70, 71].

Spectrophotometric analysis [72–74], gravimetric impactor [75], Kastle–Meyer test [76], particle sensor [37, 77], splashed area on face by magnification [78], particle counter [79–81], leucomalachite green presumptive test [10, 82], concentrations of hemoglobin in air [59, 67, 83–87], and air sampling [88, 89].

#### Particle size and composition

All systematic reviews [11, 20, 24, 25, 27, 28, 90] generation of small-sized < 5 μm particles in DMOSP. The particle size generated corresponds to droplet nuclei that could carry viruses [19]. The composition of dental aerosols differ between patients, but it consists of organic and inorganic particles.

#### Distance between two operator bays

Contamination chances are minimal in open-plan dental clinics at a distance of 5 m or more [73].

#### Blood in aerosols

Two authors [67, 87] observed that aerosols generated from electrocautery, drills, and saws in orthopedic surgeries contained hemoglobin. Even animal studies [15, 67, 85, 87, 91] confirmed Hb in aerosols. Similar results were found in human studies during oral and maxillofacial surgeries [76, 83, 84]. One of the studies even confirmed blood contamination of the internal part of the visor in 4% of cases after oral surgery procedures [76].

It was estimated that Hb inhaled by a surgeon in an hour range from 0.04 to 0.68 μg [24].

#### Contamination of personnel

Microorganisms were found on PPEs, such as sleeves, masks, face shields, and chest of the scrubs, justifying the usage of PPE to prevent further spread [26]. Contamination was evaluated with most contamination confined to the patient, operator, and assistant [72], and maximum concentration was found near the mouth of the dentist [31]. Most of the particles are concentrated ~60 cm from the patient's mouth [61].

#### HIV and other viruses in aerosols

ΦX174 phage was found in the aerosols generated after oral prophylaxis in a laboratory study [31]. Johnson et al. noted that HIV can be cultured from aerosolized particles from the bone saw, but not from te electrocautery [92]. Johnson et al. [85] estimated the inhalational risk of viruses for surgeons to be <1 HIV or 180 hepatitis B viruses per procedure.

### Characteristics of aerosols generated by various instruments

#### Micromotor and conventional air turbines

Low speeds generated the least aerosol particles, and the largest aerosol particles and the high-speed handpiece generated the greatest amount and size of splatter particles [33]. Sergis et al. [93] identified a threshold for rotation speeds for radial atomization between 80,000 and 100,000 rpm. Droplet particle size of < 5 μm was only detectable above baseline levels at revolutions > 80,000 rpm [93]. Whereas, Clarkson et al. [90] confirmed respirable aerosols when the speed is >60,000 rpm. The contamination of face masks is usually seen with high-speed rotary instruments [59].

#### Scalers

Both piezoelectric scalers and ultrasonic scalers are prone to generate a higher level of aerosols [63]. Droplet size tends to vary from 5 to 300 μm corresponding to droplet nuclei that can contain viruses [94].

#### Triplex syringe

Triplex syringe generated the largest amount of aerosols (particle size: 1.73 ± 2.23 μm) [4].

Two studies [67, 87] have compared aerosols generated by powered bone cutting tools like saws and drills and electrocautery. The aerosols generated by these procedures have been subcategorized into small particles (0.3–0.5 μm), medium particles (0.5–5 μm), and large particles (>5 μm) as explained by Sharma et al. [24].

#### Saw

The oscillating saw tends to produce the majority of medium-sized particles (56–68%) along with small-sized particles (28–40%) [24].

#### Drill

Jewett et al. [87] compared high-speed, air-powered drills, which produce 47% medium, 38% large, and 17% small particles, to high-speed drills with continuous irrigation which produce 31% medium, 59% large, and 9% small-sized particles.

#### Electrocautery

Electrocautery shows a predominance of small-sized particles both in cutting and coagulation mode. “Cutting” mode produced 90–95% small-sized particles as compared to coagulation mode with 60–78% small-sized particles [24].

### Interrater reliability

Interrater reliability was evaluated using Cohen's Kappa coefficient which came out to be 0.81 confirming a substantial level of agreement among the reviewers.

### Overall evidence regarding whether DMOSP generating bioaerosols can result in the transmission of SARS-CoV-2

There is sufficient evidence from various studies that the surgical procedures which used high powered instruments that emit or require water for cooling like ultrasonic scalers, air-water syringes, air polishing, piezo surgical handpiece, extractions using motorized handpieces, as well as bone drilling with high-speed rotary instruments (>60,000 rpm), high powered drills, oscillating saws, and electrocoagulation (cutting and coagulation mode) produce respirable aerosols which are <0.5 μm [24–28, 89, 95–97].

The patient, operator, and assistant are at the maximum risk of exposure [72] as the small particles tend to be retained in the respiratory tract. Thus, conventional masks seem insufficient against high-risk AGPs [25].

For these procedures, airborne transmission-based precautions using full PPE, procedural mitigation (high volume suction, rubber dam, preprocedural mouth rinses, and antimicrobial coolants), and 15–30 min as fallow time (the time required to allow larger droplets to settle before environmental cleaning) are required along with N95 [90]. In the operating room, the best way to decrease aerosol load is OR ventilation, with 15 air changes per hour removing 90% aerosols within 15–20 min [98, 99].

For those dental surgical procedures that use powered low-velocity instruments and may produce droplet particles > 5 μm, including 3-in-1 syringe (air-only/water-only), slow speed/electric handpiece (i.e., <60,000 rpm), surgical implant procedure, and surgical handpiece, standard PPE, and procedural mitigation without fallow time are required [90].

Finally, for those dental surgical procedures which do not use powered instruments and may produce splatter but are unlikely to produce aerosol particles < 5 μm such as tooth extraction (using forceps/elevator), manual scaling, inhalation sedation, local anesthetic administration, and standard PPE without procedural mitigation and fallow time will be sufficient [90].

In addition, there is inconclusive evidence to support the creation of infectious aerosols during DMOSP, and their potential to transmit infectious diseases like SARS CoV-2 is questionable.

## Discussion

COVID-19 as a pandemic is currently far away from being contained in a majority of countries and represents a serious potential threat to healthcare workers (HCWs), who are disproportionately affected to a higher degree during the current pandemic outbreak [23, 100]. There is uncertainty in the literature regarding the aerosol generation during DMOSP and its associated risk of viral transmission.

In this context, potential airborne transmission of SARS-CoV-2 *via* aerosols [101] as the fourth way of transmission [102] though controversially discussed is considered a significant risk [103], particularly for all those medical professionals working in close vicinity to the bronchotracheal, nasal and paranasal, oral, and oropharyngeal system [23, 104]. Furthermore, the surgeries in operation theater tend to produce splashes or sprays of body fluids that also cannot be ignored.

Although conclusive data regarding concrete numbers of incidence among dental and OMFS HCWs are lacking, some reports are indicating that dentists and OMFS are among those at elevated risk [105, 106] for transmission by patients including asymptomatic or before onset patients. In addition, considering potential aerosol transmission, due to the specific characteristics of their working environment, oral surgeons may inadvertently contribute to the cross-transmission of SARS-CoV-2 from patient to patient and HCWs by an aerosol transmission during and for some time after performing AGPs [74], especially when there is an exposure to high concentrations of aerosols in a relatively closed environment such as in surgeries [5, 105]. If it is considered highly probable of airborne transmission [5] and SARS CoV-2 is transmitted *via* aerosols [6], then the medical masks would be inadequate [5] because aerosols can both penetrate and circumnavigate masks, e.g., if compromised by moisture or if worn inadequately [5]. Face shields, too, would provide only partial protection as they leave open gaps between the shield and the HCW, and 6 feet of separation would not protect from aerosols that remain suspended in the air or are carried by air currents [5, 6].

### Do dental and maxillofacial surgical procedures generate bioaerosols (and if so, which ones), which can result in the transmission of SARS-CoV-2?

In this context, understanding aerosol transmission and its implications in dentistry is essential, as oral surgery environments with AGPs convey high risk of aerosolized transmission [23, 34], with high-speed drilling, water-air 3-1 syringe, ultrasonic scaling, and piezosurgery generally considered to be high-risk transmitters [105, 106]. In OMFS, especially tracheostomy, tracheostomy care, airway suctioning, abscess drainage, and wound irrigation (e.g., hydro-jet lavage) need to be added to this list according to the WHO recommendations [3, 107] based on prior experiences with SARS-CoV-1 [102]. Although the production of aerosols during these AGPs goes generally accepted, there is overall only weak to moderate evidence that these aerosols will in fact cause aerosol-based transmission.

In this context, it needs to be stressed that in most dental procedures suction serves as a relevant mitigation factor, reducing splatter and aerosol distribution. Chairside high-volume evacuators (HVEs) or more expensive HEPA (high-efficiency particulate arrestor) filters may reduce contamination by the operating site by 67–75% [74] to around 90% [108], or 99.7%, respectively, of particles measuring 0.3 μm in diameter [105]. Next, water or saline cooling procedures, though highly responsible for aerosolization, in turn, reduce immediate local virus load by dilution. In contrast, especially in OMFS procedures such as tracheostomy, which are usually lacking efficient suction and dilution effects, ventilation and airway-related procedures, therefore, may carry a higher risk of transmission as was shown for SARS-CoV-1 [102].

Ishihama et al. [10] assessed high-speed rotary instruments during surgery of impacted third molars and found only indirect evidence supporting the generation of aerosols during oral surgery. Even high-speed rotary instruments, and ultrasonic scalers, showed evidence of blood-contaminated droplets [109]. Comparing extraoral osteotomies in terms of orthopedic osteotomies, Nogler et al. [17, 18] confirmed contamination of OR and personnel. Even Pluim et al. [79] found moderate evidence that sawing of bone when using an oscillating saw can produce aerosols within the respirable range. Therefore, aerosol formation during OMFS bone-cutting procedures needs to be considered as a potential risk factor and the question arises whether there are potential infectious agents present in these aerosols.

### Are aerosolized airborne droplets (and to which extent is splatter) in DMOSP infective?

Particles ≤ 10 μm are considered respirable particles which are capable of reaching the lower airways, whereas particles with 10–100 μm are considered inspirable particles, i.e., limited to reaching the upper airways [102]. As viral RNA (though no viable virus) has been detected in the air associated with droplets smaller than 5 μm, the droplets may maintain infectivity [5]. SARS-CoV has been reported to travel more than six feet [110]. There is a high probability termed “beyond a reasonable doubt,” [5] that, e.g., patients' breathing, talking, and less likely coughing [102], e.g., during surgery may cause a mix of potentially infective droplets and aerosols. Microdroplets small enough to remain aloft in the air thus pose a risk of exposure at distances beyond 1–2 m from an infected patient [5], and as aerosols are estimated to travel between up to 4.5 m [111] and 27 feet (around 8 m), or room-scale [102], respectively, and stay viable for hours [112].

In this context, Klompas et al. [6] pointed out that the presence of aerosols will not automatically cause aerosol-based transmission as this depends—besides route of exposure—on factors such as the size of the inoculum, duration of exposure, and host defenses. So far, low reproduction numbers of COVID-19 (rather similar to influenza, i.e., *R*_0_ ≈ 2 as opposed to classical airborne viruses such as measles, with *R*_0_ ≈ 18) [102] indicate that either a high virus load is required, or aerosols are not the dominant mode of transmission [6].

The diameter of a mature HIV particle is 100 nm [113] and Johnson et al. [92] noted that HIV could be cultured from aerosols of a bone saw. SARS-CoV-2 is an enveloped virus ≈ 0.1 μm in diameter [114]. In an experimental study by Lee BU et al. [115] in which it was assumed that 8.97 × 10^−5^% of a respiratory fluid particle from a patient with COVID-19 is occupied by SARS-CoV-2, hence the minimum size of a respiratory particle that can contain SARS-CoV-2 is calculated to be ~9.3 μm. If the patient supposedly has a high viral load, then it can decrease the minimum size of respiratory particles containing SARS-CoV-2, thereby increasing the probability of aerosol generation of the viruses [115]. It was found that the virus SARS-CoV-2 was viable even after 3 h, with limited loss of viability [116].

This is of utmost importance, as the presence of SARS-CoV-2 is also reported in particles ranging between 0.25 and 1.0 μm [112]. Thus, theoretically, a bioaerosol carrying viruses might remain within the proximity of the dental chair even after the patient leaves.

Severe acute respiratory syndrome coronavirus 2 (SARS-CoV-2) has been found in infected saliva [117] thus local virus load also explains discussion on preprocedural mouth rinsing (e.g., chlorhexidine CHX), which leads to a mean reduction of 68.4% colony-forming bacterial units in dental aerosols [117]. It has been proven to be efficient against several infectious viruses [105]. However, there is currently no evidence for the use of hydrogen peroxide mouth rinsing [118, 119], even though its use was initially publicized [23, 120, 121].

According to recent Cochrane reviews, there is currently no evidence yet relating to the benefits and risks of healthcare workers using antimicrobial mouthwashes or nasal sprays to protect themselves from contracting COVID-19 [122, 123]. Nevertheless, it should always be kept in mind that airborne transmission *via* aerosols remains an imponderable threat, especially to oral surgeons even though so far un-proven [102] and still speculative. This uncertainty may be because it is difficult to detect contaminated air, as infectious aerosols are usually extremely dilute, and it is hard to collect and culture fine particles [124]. As aerosol transmission is classified as obligate, preferential, or opportunistic, based on the agent's capacity to be transmitted and to induce the disease through fine-particle aerosols and other routes [124, 125], an opportunistic transmission potential should be assigned to SARS-CoV-2 [5].

### Is additional standard personal protective equipment essential to prevent the spreading of SARS-CoV-2 and thus COVID-19 during aerosol-generating DMOSP?

Standard local disinfection and decontamination protocols plus pandemic-adapted distancing procedures should always be ensured as a basic principle. There have been many recommendations from respective governmental or health service institutions of different countries for HCWs, and many recommendations are heterogeneous and epidemiological data relative to their effectiveness against COVID-19 are limited [126].

Therefore, from a clinical point of view, the most contingent question arises as to which is an adequate/appropriate PPE for DMOSP and whether this question can be answered from an evidence-based point of view. All OR personnel should be considered contaminated after each procedure and PPE should be preferred with well-established donning and doffing practices [24]. However, to save resources, PPE can be chosen depending on the planned procedure and the infection status of the patient [23].

So far, according to consensus, power air-purifying respirators (PAPR), which were scarcely available during the outbreak, have not been considered mandatory to safely avoid aerosol-borne transmission in OMFS [23]. At present, N95/FFP2 for AGPs and N99/FFP3 masks with valves [23] for surgery in infected patients, respectively, are most frequently recommended, instead [127–131].

Chu et al. [132] concluded in their systematic review regarding the spread of viruses *via* aerosols, that respirators would be more protective than medical masks alone. Even another systematic review by Sobti et al. [25] has confirmed that conventional masks do not offer protection against high-risk AGPs. This is in contrast with a study by Bartoszko et al. [126] regarding the use of medical masks vs. N95 respirators in preventing laboratory-confirmed viral infection and respiratory illness specifically in HCWs, analyzed four RCTs including coronavirus and concluded that the use of medical masks did not increase the rate of laboratory-confirmed respiratory infection (OR 1.06) or clinically respiratory illness (OR 1.49).

Nevertheless, at least for AGPs, N95 respirators/FFP-2 masks at present are unanimously recommended by national and international guidelines. The underlying rationale most probably relates to the high level of viral exposure from droplet clouds rather than transmission by the airborne route [102, 133], but is also due to the conspicuous lack of understanding of the detailed mechanisms of SARS-CoV-2 transmission, which may also explain the discrepancy of the recommendation to protect the HCWs with surgical masks vs. respirators.

Accordingly, there is inconsistency in recommendations for routine care and non-AGPs of COVID-19 [5] as the WHO, Public Health England, and the Public Health Agency of Canada recommend the use of medical/surgical masks for non-AGPs [107, 134, 135] in contrast to several societies and national associations recommending N95/FFP2 also for non-AGPs over the less expensive and more readily available medical masks [126].

According to Zimmermann and Nkenke [23], for routine care of low-risk patients (i.e., symptoms-free), the use of medical masks and gloves to protect against droplet transmission is considered sufficient.

As a consequence, at least under pandemic conditions, to save resources [23] and according to available evidence as presented in this article, it may seem reasonable to differentiate between low-risk and high-risk dental and OMFS procedures, with just the latter ones requiring special precautions to prevent droplet and especially aerosolized disease transmission. For low-risk treatments, current empirical data and the absence of clear scientific evidence for aerosol transmission of SARS-CoV-2 provide sufficient rationale for the use of surgical masks, which in analogy should apply in DMOSP.

There are several limitations of this review: (1) The included studies did not directly study an association between potential aerosolization during DMOSP and SARS-CoV-2. (2) Until now, there is no evidence comparing surgical masks vs. respirators regarding SARS-CoV-2 transmission, as all studies so far available dealt with other viruses (influenza and SARS-CoV-1 viruses) rather than SARS CoV-2. Thus, as there was no indirectness and extreme heterogeneity in the included studies, the confidence of evidence for this review must be rated as very low-quality evidence. (3) As a consequence, any evidence provided by the current review was gathered by extrapolation from available experimental and empirical evidence not based on SARS-CoV-2. The results of the present review, therefore, should be interpreted with great caution.

## Conclusion

As there is laboratory experimental evidence supporting that dental and OMF aerosol transmission of the viable virus is possible, the risk for SARS-CoV-2 transmission from dental and OMF AGPs, therefore, needs to be confirmed through isolation and culture of viable virus in the clinical environment. At present, according to available very weak/inconclusive evidence, the transmission of SARS-CoV-2 *via* infective aerosol during AGPs, so far, must remain speculative and controversial. As, however, this is a probable opportunistic way of transmission which cannot be sufficiently excluded and therefore should not be dismissed out of hand prematurely, proper and equally important properly applied protective equipment (i.e., N95 respirators or FFP-2 masks or above regarding mouth and nose protection) should always be used during AGPs. Last but not least, there is an urgent need for studies comparing respirators to surgical masks during dental and OMFS AGPs for protection against SARS-CoV-2 transmission.

## Data availability statement

The raw data supporting the conclusions of this article will be made available by the authors, without undue reservation.

## Author contributions

All authors have read and approved the final version of the manuscript.

## Conflict of interest

Author AN was employed by University Hospital Marburg UKGM GmbH. The remaining authors declare that the research was conducted in the absence of any commercial or financial relationships that could be construed as a potential conflict of interest.

## Publisher's note

All claims expressed in this article are solely those of the authors and do not necessarily represent those of their affiliated organizations, or those of the publisher, the editors and the reviewers. Any product that may be evaluated in this article, or claim that may be made by its manufacturer, is not guaranteed or endorsed by the publisher.
