# Supplementary material for: Can aerosols-generating dental, oral and maxillofacial, and orthopedic surgical procedures lead to disease transmission? An implication on the current COVID-19 pandemic
Source: Front Oral Health. 2022 Aug 1;3:974644. doi: 10.3389/froh.2022.974644 (PMC9376374; doi:10.3389/froh.2022.974644)
Supplement: Supplementary file 1 [file Table_1.docx]

| **AUTHORS AND YEAR** | **TYPE OF STUDY** | **HOW OUTCOMES WERE MEASURED** | **TYPE OF SURGICAL PROCEDURES** | **TYPE OF SURGICAL INSTRUMENTS USED** | **CONCLUSION** | **EVIDENCE OF AEROSOL GENERATION** | **EVIDENCE OF TRANSMISSION RISK** | **MICROBIAL SPECIES** |
| --- | --- | --- | --- | --- | --- | --- | --- | --- |
| Putzer D et al,2021 [30] | Laboratory study (Porcine knee) | Petri dishes with Columbia blood agar | Resection of cartilage, cortical and cancellous bone | -High Speed Drilling system | The highest contamination was found perpendicular to the bur rotation axis in a distance 0.5 m from the bur, at a height of 1.4 m. Around this spot, colony-forming units count isotropically dropped to less than 100 CFUs at an area of 0.5 m in diameter | Clear evidence supporting aerosol generation | Unclear | Staphylococcus aureus bacteria |
| Sergis A et al, 2021 [31] | Laboratory study  (a modified dental training mannequin) | Laser beam for illumination and photographic analysis | Enamel cutting | Micromotor  Conventional air turbine | Complex structured sprays were produced by water-cooled rotary instruments, which, in the worst case of an air turbine, included droplet projection speeds in excess of 12m/s and the formation of millions of small droplets that may remain suspended. | Clear evidence supporting aerosol generation | Unclear | N/A |
| Han P et al,2021 [4] | Laboratory study (Mannequin) | Sodium fluorescein, filter paper and fluorescent microscope | Mock dental procedures | ultrasonic scaler,triplex syringe, high-speed and low-speed handpiece | At 120 cm away from the source, the high-speed handpiece generated the greatest amount and size (656 ± 551 µm) of splatter particles, while the triplex syringe generated the largest amou6nt of aerosols (particle size: 1.73 ± 2.23 µm) | Clear evidence supporting aerosol generation | Clear evidence of transmission risk  All four dental AGPs need to  be carefully considered in the context of planning risk-based additional precautions to prevent  potential airborne transmission. | N/A |
| Llandro H et al.,2021 [32] | Laboratory study (Mannequin) | digital image analysis and spectrofuorometric analysis | Orthodontic debonding | Speed-increasing handpiece | Contamination across the eight-metre experimental rig was 3% of the positive control on spectrofluorometric analysis and 0% on image analysis. Contamination of the operator, assistant and mannequin was 8%, 25% and 28% of the positive control, respectively. | Clear evidence supporting aerosol generation | Unclear | N/A |
| Holliday R et al, 2021 [33] | Laboratory study (Mannequin) | image and spectrofluorometric analysis | Crown preparation | high-speed air-turbine handpiece | Aerosols produced by dental procedures have the potential to contaminate distant sites and the majority of settled aerosol is detectable after 10 min | Clear evidence supporting aerosol generation | Cross-infection risk from dental procedures in an open plan clinic appears small when bays are ≥ 5 m apart | N/A |
| Liu Z et al.,2021 [34] | Laboratory study | Agar plates | Oral prophylaxis | Not mentioned | Highest bioaerosol concentration was located near the mouth of the dentist. At the equipment trays, the two kinds of bioaerosols deposition rates per unit area were as high as 10.5% and 6.6%, respectively | Clear evidence supporting aerosol generation | Unclear | Serratia marcescens and ΦX174 phage |
| Takanabe Y et al.,2021  [35] | Laboratory study | laser lights and a high-sensitivity camera | N/M | Micromotor  Portable Vivace unit | The results indicate that aerosols tend to scatter upward immediately after generation and then gradually disperse into the surroundings. Most particles are less than 5 µm. | Clear evidence supporting aerosol generation | Unclear | N/A |
| Graziani F et al,2021 [36] | Cross-sectional  (Human study) | gravimetric impactor | ultrasonic scaling | Ultrasonic scaler | The 0.3-µm particles showed non-significant increases at 15/30 min. The 0.5–1-µm particles increased at EI (p < 0.05), and 0.5 µm remained high for another 15 min. Overall, all submicron aerosol particles showed a slow decrease to normal values. Particles measuring 3–5 µm showed non-significant increases at EI. Particles measuring 10 µm did not show any increases but a continuous reduction (p < 0.001 versus 0.3 µm, p < 0.01 versus 0.5 µm, and p < 0.05 versus 1–3 µm) | Clear evidence supporting aerosol generation | Unclear | N/A |
| Sobti A et al.,2021 [25] | Systematic review | Systematic review | Orthopedic (lumbar spine, robotic, revision hip arthroplasty, total knee arthroplasty, cervical spine) | High speed cutter, electrosurgical apparatus, domestic electric drills, ultrasound device, total hip arthroplasty instruments, power surgical drills | Most orthopedic procedures are high-risk aerosol generating procedures (AGPs). Conventional surgical masks do not offer protection against high-risk AGPs. | Clear evidence supporting aerosol generation | Clear evidence | Bacterial |
| Mirabod P et al., 2021 [37] | Laboratory study | Illumination and photographic analysis (Shadowgraphy) | Scaling | Ultrasonic scalers | The droplet size varies from 5 µm to 300 µm: which corresponds to droplet nuclei that could carry viruses | Clear evidence supporting aerosol generation | Clear evidence | N/A |
| Nóbrega MTC et al.,2021 [26] | Scoping review | Scoping review | Routine Dental procedures | High speed dental equipment, ultrasonic scaler, piezoelectric scaler, mechanical scaling and triple syringes | Total particle concentration are higher during grinding, followed by drilling, polishing, scaling and finally other general dental procedures | Clear evidence supporting aerosol generation | Unclear | Bacterial, volume of aerosols |
| Sharma et al.,2021 [24] | Systematic review | Systematic review | Orthopaedic procedures | Burr/Cutter/Ultrasonic devices  o Saw  o Dril  o Irrigator/Hydro-debridement devices  o Electrocautery | The  pooled estimate of particle density was 390.74 mg/m3, Total Particle Count, 6.08 _ 106/m3, and Microbial  Air Contamination, 8.08 CFU/m3. Small sized particles (</ ¼ 0.5 mm) were found to be 37 and 1604 times  more frequent in the aerosol cloud in comparison to medium and large sized particles respectively. | Clear evidence | there is evidence to suggest that OSPs generate large amounts of bioaerosols, their  potential to transmit infectious diseases like COVID-19 is questionable | Bacteria |
| Ionescu AC et al,2020 [38] | Laboratory study (mannequin) | Mitis salivarius-bacitracin agar plates | Cavity preparation and scaling | Air tubine handpiece, contra-angle handpiece and ultrasonic scaler | The maximum distance of tracer detection was 360 centimeters for air turbine, 300 cm for contra-angle handpiece, and 240 cm for ultrasonic scaler | Clear evidence supporting aerosol generation | Clear evidence confirming pattern and degree of contamination were related to the distance from the infection source, with concerns for the airborne spreading of the pandemic disease agents | Streptococcus mutans |
| Allison JR et al., 2020 [39] | Laboratory study  (mannequin) | Photographic image analysis and spectrofluoro-metric analysis | - Anterior crown preparation - Full mouth scaling | High-speed air-turbine, ultrasonic scaler and 3-in-1 spray | - dental procedures have potential to deposit aerosol and splatter at some distance from the source, being effectively cleared by 30 minutes in our setting | This study provides moderate evidence of aerosol generation by high-speed air-turbine and ultrasonic scaler | Unclear | N/A |
| Clementini M et al,2020 [27] | Narrative review | Narrative review | Unclear | Rotary instruments, ultrasonic scalers, air polishers and air abrasion unit | -confirmation of surface contamination and provided the insight pf Sars Cov-2 detection in the air, particularly in indorr settings with poor ventilation where aerosol generating procedures are performed | Clear evidence supporting aerosol generation | Clear evidence | N/A |
| Innes N et al., 2020 [28] | Systematic review | Contamination levels for different dental procedures (microbial surrogate measures mainly oral microbiota and blood or blood-stained water)  () | Tooth extraction; scaling and polishing | - Ultrasonic scaler (uss), high-speed air-rotor (hsar) in oral surgery   Slow-speed handpiece, air-water (triple) syringe, air-polishing in prophylaxis and hand scaling | - there was evidence of contamination of surfaces around the surgery environment/ personnel or contamination in air from all procedures which were assessed | - there is higher risk (uss, hsar, air-water syringe [air only or air/water combined], air  Polishing, extractions using motorized handpieces)  - there is moderate risk (slow-speed handpieces, prophylaxis with pumice, extractions)  - lower risk (air-water syringe [water only] and hand scaling | High and moderate risk | Bacterial |
| Clarkson JRC et al., 2020 [40] | systematic review | Narrative review | Any operative surgical procedures performed by high and low speed handpieces  Scaling using ultrasonic scaler | - high speed air/electric rotor (i.e. >60,000 rpm)  - slow speed/ electric handpiece (i.e. <60,000 rpm)   - Piezo surgical handpiece - Air polishers - 3-in-1 syringe (air and water combined) | - those dental surgical procedures which use powered, high speed instruments that emit or require water or irrigation for cooling and will produce aerosol particles ≤ 5 μm such as tooth extractions when using motorized high-speed handpieces, ultrasonic scalers, 3-in-1 syringes, piezo surgical handpiece and bone drilling using high speed rotary instruments (high speed air/electric rotor, i.e. Speed >60,000 rpm) produce respirable aerosols | High risk when using high speed air/ electric rotor  (i.e. >60,000 rpm) and scaling using ultrasonic scaler | High risk and low risk | Bacterial |
| Aguilar Duran et al.,2020 [41] | Cross sectional  (Human study) | Kastle-Meyer test (phenolphthalein) | Oral surgery procedures ( extraction of impacted or erupted teeth or dental implant placement) | for tooth extraction, high-speed air-turbine handpiece with water cooling or low-speed electric straight handpiece with external cooling using a syringe; for implant placement, electric contra-angled handpiece with saline cooling incorporated in the handpiece | The risk of clinician contamination with blood during tooth extraction and implant placement was 46%. The risk increased with the use of high-speed instruments and longer surgery time | The authors observed blood contamination in all types of procedures, and in 4% of the cases, the internal part of the visor was also affected | Clear evidence | N/A |
| Gund et al.,2020 [42] | Cross-sectional (Human study) | Agar plates and Bacteria were identified by MALDI TOF mass spectrometry | carious cavity preparation, tooth substance preparation, trepanation and root canal treatment, supragingival ultrasonic application and subgingival periodontal ultrasonic instrumentation | High speed and medium speed handpiece with water cooling, ultrasonic instruments | All masks and all gloves used during treatment displayed bacterial contamination | Surgical masks are contaminated after aerosol-producing dental treatment procedures. Used masks have a potential to be a source of bacterial contamination of the hands | Clear evidence | Streptococci spp. and Staphylococci spp. representing the oral and cutaneous flora dominated. |
| Zemouri et al.,2020 [43] | Cross-sectional (Human study) | Petri dishes with blood agar | Active and passive sampling  Procedures not defined | N/M | The samples contained up to 655 CFU/plate/30 minutes and 418 CFU/m3 /30 minutes during dental treatment for active and passive sampling, respectively. The level of contamination after treatment and at 1.5 m distance from the patient’s head was similar to the start of the day. | Contamination in dental clinics due to aerosols is mainly low, although high level of contamination with taxa from both human and water origin was found within 80 cm around the head of the patient | Clear evidence | Bacteria |
| Morris et al.,2020 [44] | Prospective  human study  (RCT) | Particle Counts (different  sizes), and culture plates | Shoulder arthoplasty | N/M | Significantly lower CFUs in the localized laminar air  flow device group, after adjusting for number of OR  personnel and surgical time | Clear evidence | Unclear | Bacteria |
| Matys J et al.,2020 [45] | Laborartory study (Manikin ) | Particle Sensor | Cavity preparation, scaling | High speed handpiece  Low speed handpiece,  Ultrasonic scaler | Caries removal at high-speed handpiece and saliva ejector generates the highest amount of spray particles. | Clear evidence supporting aerosol generation | Unclear | N/A |
| Kirschbaum et al.,2020 [46] | Prospective  human study  (randomized  cohort study) | Particle Counts (different  sizes), Swabs taken from ‘safe  air smoke evacuator’ for  microbial cultures | Total knee arthoplasty | N/M | The use of a LAF system signifcantly reduces the particle load and therefore potential bacterial contamination regardless of the time or place of measurement and therefore seems to be a useful tool for infection prevention | Clear evidence | Unclear | Bacteria |
| Anis et al., 2019 [47] | Prospective human design (RCT) | Particle Sensor and blood agar plates | Total joint arthoplasty | N/M | a C‐UVC air disinfection and recirculation unit led to a significant reduction in both TPC and VPC and a non‐significant reduction in CFU | Clear evidence | Unclear | Bacteria |
| Divya R et al.,2019 [48] | Cross sectional (human study) | Bacterial cultures for Colony forming units | Minor oral surgical procedures like impaction, transalveolar impactions and alveoloplasty | High speed handpiece | Aerosol and splatter contamination analysed by bacterial CFU exceeded permissible limits | Clear evidence supporting aerosol generation | Clear evidence | Bacteria |
| Watanabe A et al 2018 [49] | Clinical study (cross sectional) | ATP bioluminescence, Mitis salivaris agar | Cavity preparation and scaling | Ultrasonic scaler, high speed turbine/micromotor | Maximum contamination was found on the patient’s googles. | Clear evidence supporting aerosol generation | Clear evidence | Bacterial |
| Kobza et al.,2018 [50] | Cross sectional | Gram staining and Malt Extract Agar | N/M | N/M | Quantitative and qualitative evaluation showed that during treatment, there is a significant increase in airborne concentration of bacteria and fungi | Clear evidence supporting aerosol generation | Exposure to the microorganisms identified is not a significant occupational hazard for dental care professionals; however, evidence-based prevention measures are recommended | Bacterial and fungal |
| Nunes et al.,2018 [51] | Cross sectional | Sabouraud Agar | 1, inside the X-ray room; 2, next to the portable revelation box; 3, in the central corridor that access the dentist room; 4, next to dental cuspidor; 5, next to sink for instrument washing; 6, next to the operator; and 7, next to the handwash sink. | N/M | In teaching clinics, this contamination is greater due to the high number of occupants and procedures performed at the same time | Clear evidence supporting aerosol generation | Clear evidence | Fungal |
| Pluim JME et al., 2018 [52] | Autopsy study | Airborne particles were counted per diameter by a particle counter | Sawing in bone | oscillating saw | - increasing the saw blade frequency or decreasing the saw blade contact load resulted in a higher production of aerosol bone dust.  - in order to limit bone aerosol production when using oscillating saws, one should try to keep the saw blade frequency as low and saw blade contact force as high as possible within the limits of safety and practicality. | Moderate Evidence that sawing of bone e.g. during autopsy using an oscillating Saw can produce aerosols within  the respirable range. | Unclear | N/A |
| Adhikari A et al,2017 [53] | Cross-sectional | Bacterial counts on Trpticase Soy agar | Dental cleaning procedures | N/M | The concentration of airborne bacteria did not increase significantly during the cleaning procedures | Clear evidence of aerosol generation | Unclear | Bacterial |
| Putzer D et al 2017 [20] | Cadaveric study | Bacterial counts on mannitol salt agar | Lumbar spine  Hydro surgery debridement | N/M | Contaminated aerosols spread over whole surgical room and contaminate the theater and all personnel. | Clear evidence of aerosol generation | Unclear | Staph aureus |
| Singh A et al., 2016 [54] | Comparative study  (human) | Bacterial counts on agar plates | scaling | ultrasonic scaler | bacterial counts were highly significant when compared before and during the treatment | Unclear | Weak evidence that high levels of environmental bacterial contamination is created following ultrasonic scaling | Bacterial |
| Wendlandt et al.,2016 [55] | In vitro study (artificial foam bone) | Ultraviolet illumination | Hip and knee arthroplasty | Tools used in arthroplasty | concluded that the contamination risk was 30%  while wearing conventional clothing whereas none of the 20  subjects using the surgical helmet system reported any  contamination after removal of the protective clothing | Clear | Clear | NA |
| Veena HR et al., 2015 [56] | In vitro study | Ultrafiltrate-  Containing fluorescent dye | scaling | ultrasonic scaler | contamination was found up to 4ft from the ‘patient’ and the ‘aerosol’ cloud remained in the operatory air from 0-30 min after the procedure was completed | Very weak evidence that aerosols are  Produced following ultrasonic  Scaling | Unclear | Bacterial |
| Jimson S et al,2015 [57] | Cross sectional (human) | Bacterial counts on agar plates | Mandibular third molar impaction | N/M | Average values of CFU/cm2 were high in the plates exposed after surgery | Clear evidence of aerosol generation | Unclear | Bacterial |
| Chuang CY et al.,2014 [58] | Human case series | Tryptic soy  agar | scaling | Ultrasonic sCcaler | Bacterial aerosols contamination could spread a horizontal distance of 100 cm and a vertical distance of 50 cm from a patient’s oral cavity, and remain  airborne suspended for 20 minutes | Clear evidence of aerosol generation | Clear evidence | Bacterial |
| Polednik,2014 [59] | Cross sectional  (humans) | Petri dishes with Tripticase Soy Agar for bacteria and Sabourand Dextrose Agar with chloramphenicol for fungi | Drilling, Grin ding, Polishing, Scaling, Restorations, Extractions | N/M | The highest submicrometer particle concentrations were observed during dental grinding and they were on average 16 times higher than the indoor background. Certain metallic trace elements and total carbon concentrations were significantly elevated (410 times) in the particles deposited in the operating room | Clear evidence | Dental procedures also contributed to increased bacterial contamination that may pose a health risk both for dental personnel and patients | Bacteria and fungi |
| Nejatidanesh et al.,2013 [60] | Cross sectional (humans) | Splashed area on face shield by magnification | Crown preparation and scaling | ultrasonic scaling unit and tooth preparation with high‑speed hand piece for fixed prosthesis as the prosthetic treatment | The areas around nose and the inner corner of eyes were the most contaminated areas. Zygoma was the least contaminated area. The contaminated areas during periodontal treatments were significantly more than prosthetic treatments (P < 0.05). There was no significant difference between contamination on left and right sides of the face | Clear evidence | During dental practice, central areas of the face such as inner part of the eyes and around the nose were most contaminated areas. These parts are the important areas for transmission of infection | N/A |
| Manarte-Monteiro et al.,2013 [61] | Cross sectional (human) | Bacterial counts on blood agar | Dentistry and endodontic procedures | Manual instruments, high speed handpieces (turbine) with water cooling | Aerosols’ CFU counts were significantly higher at 0.5m and during endodontic treatments. Longer treatment times were associated with higher CFU counts both in dentistry and endodontic procedures. The use and time of turbine use did not significantly affect the CFU counts | Clear evidence | Unclear | Bacterial |
| Yen-Tseng et al.,2013 [62] | Cross-sectional (human) | CMRASII plates to detect MRSA | Periodontal procedures | Ultrasonic scalers and surgical procedures using high speed handpieces | No MRSA colonies at 24-48 hours, and only few individuals had methicillin resistant coagulase negative staphylococci | Clear evidence | The occupational risk of MRSA infection in a dental setting is probably minimal | Bacterial |
| Pasquarella et al.,2012 [63] | Prospective cohort study | Bacterial counts on agar plates | Conservative dentistry | Ablator, air water syringe, micro engine, turbine | Microbial air contamination was highest during dental treatments, and decreased significantly at the end of the working activity (pb0.05). The microbial buildup on surfaces increased significantly during the working hours. | Clear evidence | Unclear | Bacterial |
| Guida M et al.,2012 [64] | Cross sectional (human) | Bacterial counts on agar plates | Conservative and oral surgery practices | N/M | Decrease in air contamination was seen in the end of the day | Clear evidence of aerosol generation | Unclear | Bacterial |
| Pereira ML et al 2012 [65] | Human study | Portable particle counter | Orthopaedic | Cleaning/moving the patient Use of electrosurgical apparatus Movement of the surgical team) | Most of the events generating aerosol particles in an orthopaedic surgery room are brief,intermittent and highly variable | Clear evidence of aerosol generation | Unclear | N/M |
| Kucukdurmaz F et al,2012 [66] | Laboratory study | Portable particle counter and bacterial counts on blood agar | Orthopedic | Domestic electric drills (DED) | DED produced statistically significant higher levels of particles than the ambient air. Bacterial growth were found in the exhaust air of all running drills | Clear evidence of aerosol generation | Clear | Bacterial |
| Labaf et al.,2011 [67] | In- vitro study | Blood agar plates | Scaling, tooth preparation, access cavity preparation | Ultrasonic scaling, high speed dental handpiece | Greatest amount of aerosol was seen in prosthodontic treatment and least value was shown in endodontic treatment | Clear evidence | Unclear | Micro-organisms (Not specified) |
| Yamada H et al., 2011 [68] | Comparative study  (human) | Extraoral evacuator | - Third molar extractions - Full-crown preparation - Inlay cavity preparation,   Scaling | - High-speed rotary instruments - Ultrasonic scaler | - the higher proportion of positive tests following 3^rd^ molar extraction was at 50 cm, and 100 cm  - dental procedures using high-speed instruments can result in blood spread  Up to 100cm around the patient and into the breathing zone of the dental staff | Clear evidence of generating droplets contaminated with blood rather than small aerosols | Unknown | N/A |
| Ishihama K et al.,2009 [69] | Clinical human case series | leucomalachite green presumptive test | - impacted mandibular third molars | - high-speed rotary instruments | At locations 20 and 100 cm from the surgical site, 76% and 57%, respectively, of the particulates were positive in blood presumptive tests. | direct evidence supporting generating aerosols during oral surgery | These results indicate the risk of cross-infection at the dental practice | N/A |
| Dutil et al.,2009 [70] | Cross sectional | Culture-based method (R2A, and blood agar with and without O2) and fluorescence microscopy | - Scaling | - Ultrasonic scaling | dental staff and patients were exposed to up to 1.86 E+05 bacteria/m3 generated during treatment | Clear evidence | The small diameter of the aerosols generated suggests that the risk of contact between the aerosolized bacteria and the respiratory system of exposed individuals is likely to occur | Bacterias |
| Ishihama K et al.,2008 [8] | Clinical human case series | leucomalachite green presumptive test | - impacted mandibular third molars | - high-speed rotary instruments | dental procedures with high-speed instruments exposed surgeons to possible bloodborne infections by splatters in nearly 90% of the cases. more than 50% of the stains were invisible to  the naked eye | indirect evidence supporting generating aerosols during oral surgery | unclear | N/A |
| Pina-Vaz et al.,2008 [71] | Cross sectional (Human study) | Blood agar plates | - access cavity preparation | - High speed hand piece | There were significant statistical differences between the number of cfus before and after the dental procedure in the plates next to the patient. Between the three groups, the differences were not statistically significant. Contamination in the plates away from the patient, showing that bacterial aerosols will also settle a long distance from the patient after the conclusion of the procedure. | Clear evidence | Clear evidence | Bacterial |
| Sotiriou et al.,2008 [72] | Cross sectional (human study) | Particle count | - Drilling | - N/M | The dental procedures produced number concentrations of relatively small particles (0.5 μm). Also, these dental procedures caused significant elevation above background of certain trace elements (measured by X-ray fluorescence) but did not cause any elevation of elemental carbon (measured by thermal optical reflectance). Dental drilling procedures aerosolize saliva and products of drilling, producing particles small enough to penetrate deep into the lungs | Clear evidence | Clear evidence | N/A |
| Azari MR et al,2008 [73] | Human study | Bacterial colonies | - routine dental treatment | - N/M | Staph sps were found in some areas of dental school suspecting it as hazard | indirect evidence supporting generating aerosols during oral surgery | unclear | Bacterial and fungal |
| Cristina ML et al.,2008 [74] | Human study | Concentration of Hb in the air | - Dental operations (apicectomies,premolar, and third molar extractions | - N/M | Contamination of air and surfaces by blood particulates were found to be positive | Clear evidence | Unclear | N/A |
| Grecio PM et al., 2008 [75] | Human study | Dark field microscopy, gram stain microscopy and chemical identification | - Orthodontic debonding | - Standard debonding pliers | Contamination is positive | Clear evidence | Unclear | Bacterial |
| Perdelli F et al,2008 [76] | Human study | Concentration of Hb | - Autopsy, oral and maxillofacial operations and dental treatments | - N/M | Haemoglobin was detected in 38.64% of the samples | Clear evidence | Unclear | N/M |
| Szymańska J, 2007 [11] | Narrative review | Narrative review | - Scaling and cavity preparartion | - ultrasonic scaler tip and a bur on a high-speed handpiece | The most intensive aerosol and splatter emission occurs during the work of an ultrasonic scaler tip and a bur on a high-speed handpiece | Clear evidence | Unclear | Bacterial |
| Motta et al.,2007 [77] | Cross sectional | Culture media plates | Dental procedures | N/M | An increase in the number of microorganisms was observed during clinical procedures | Clear evidence | Unclear | Bacterial |
| Shivakumar et al.,2007 [78] | Prospective cohort study | Culture media plates | - Scaling, restorations | - Ultrasonic scaling, micromotor handpiece and air rotor handpiece | The results showed that atmospheric microbial contamination (CFUs/plate) was 4 times higher during working sessions as compared to the levels before the working sessions. At the end of the working day, aerosols decreased by almost 3 times that seen during work | Clear evidence | Clear evidence | Bacterial |
| Rautemaa R et al., 2006 [79] | Comparative study  (human) | Settle plates | - Restorative dentistry - Periodontal and orthodontic treatment | - High-speed rotary instruments - Ultrasonic scaler | - high-speed and ultrasonic dental instruments lead to substantially greater environmental contamination than procedures which don’t.  - the facial masks became equally contaminated during the use of high-speed rotating instruments | Weak  Evidence that these procedures  Generate small inhalable aerosols | Unclear | Bacterial |
| Timmerman MF et al.,2004 [80] | Human study cross sectional | Culture media plates | - Scaling | - Ultrasonic unit | Only limited atmospheric microbial contamination is produced when using a piezoelectric ultrasonic scaler | Clear evidence | Clear evidence | Bacterial |
| Prospero et al.,2003 [81] | Cross sectional | Culture media plates | - Restorative and scaling | - N/M | Most particles have diameters of less than 5 µm and are concentrated within approximately 60 cm of the patient’s mouth | Clear evidence | Clear | Bacterial |
| Nogler M et al., 2003 [16] | Cadaver study | Culture media plates | - Hip arthroplasty | - High-speed cutting device - Ultrasound device | - this study provides moderate evidence of significant and widespread contamination of the theatre via the airborne route using a high-speed cutter compared to an ultrasound device.  - the level of contamination was significantly higher using the high-speed cutter compared to the ultrasound device | Both the ultrasound and the high-speed cutter produced aerosols which covered the whole operating theatre and all personnel present during the procedure. | Infectious agents may be present in these aerosols | Bacterial |
| .Nogler M et al., 2001a [17] | Cadaver study | Culture media plates | High sped cutting in lumbar spine surgery in laminectomies | High-speed cutting device with a 6 mm ball cutter | - the use of high-speed cutters in spinal surgery produces an aerosol that can be contaminated with blood-borne pathogens from infected patients. This aerosol is spread over the whole surgical room and contaminates the room and all personnel present. | The study provides moderate  Evidence of aerosol generation  During procedures using high-speed cutting devices. | NA | Bacterial |
| Nogler M et al., 2001b [18] | Cadaver study | Surveillance cultures | - High-speed cutting in - Cervical spine surgery | - High-speed cutting device   With a 6 mm ball cutter | - the study showed that the use of high-speed cutters in surgery of the cervical spine produces an aerosol cloud that is spread over the whole surgical room and contaminates the theater and all personnel present | The study provides moderate  Evidence of aerosol generation  During procedures using high-speed cutting devices | NA | NA |
| Toroglu et al.,2001 [82] | Case control (split mouth design) | Blood agar plates | Debonding | Air turbine handpiece | Orthodontists are exposed to high levels of aerosol generation and contamination during the debonding procedure, and preprocedural chlorhexidine gluconate mouth rinse appears to be ineffective in decreasing the exposure to infectious agents | Clear evidence | Unclear | Bacterias |
| Kedjarune U et al.,2000 [83] | Human comparative study | Slit-to-Agar air sampler | - Endodontic - Scaling - Cavity preparation | - ultrasonic scaler and high speed drill | The concentration of total bacterial aerosols and Bacillus sp. in air which circulated in the dental clinic was lower at the end of the day than at the beginning | Clear evidence of aerosol generation | Unclear | Bacillus sps |
| Bennett AM et al., 2000 [84] | Clinical human case series | Microbial air sampling | - Cavity preparation - Scaling | - sonic and ultrasonic scaler | - this study suggests that peaks in bacterial airborne contamination are more likely to occur during sonic and ultrasonic scaling but overall due to this study’s limited presentation of data, its findings are deemed to be inconclusive.  - blood was not detected in any of the personal air samples | Weak evidence | Unclear | Bacterial |
| Harrel SK et al 1998 [85] | Laboratory study on mannequin (comparative study | Fluorescein dyes and UV light | - Scaling | - Ultrasonic scalers - Gracey curettes | When compared to hand scalers, ultrasonic scalers produced significant aerosol and splatter | Clear evidence of aerosol generation | Unclear | N/M |
| Johnson et al.,1997 [86] | Animal (canine)  study | Particle concentration, particle  size, hemoglobin content in  aerosol. | Characterized blood containing aerosols during canine THA | - Oscillating power saw | Mean particle count was 5.45 _ 108 ± 3.54 _ 108.  Mass median diameter was 0.89 mm. Mean count  median diameter was 0.18 mm. Mean Hb aerosol  mass was 133 ng | Clear evidence | Clear | Viral |
| Grenier D,1995 [87] | Clinical human case series | Trypticase soy agar plates | - Operative treatments - Ultrasonic scaling | high-speed dental drill and  ultrasonic scaler | The maximum levels of air contamination in the closed dental operatory were observed while dental treatments were being performed (four trials; 216 6 75 CFU/m3 for ultrasonic scaling treatments and 75 6 22 CFU/m3 for operative treatments). | Clear evidence of aerosol generation | Unclear | Bacterial |
| Yeh et al.,1995 [88] | Human study | Concentrations of different  sizes of particles, at different  stages of surgery | 5 THAs, 3 TKAs, 1 back fusion, 1 hip   - reconstruction | Bone drill, electrocautery, scalpel | Highest amount of aerosol was generated by  electrocautery and irrigation-suction. Room clean  up did not increase aerosol concentration | Clear evidence | Unclear | N/M |
| Yeh et al.,1995 [15] | Animal study | Aerosol mass and RBC  concentration | - Total hip replacement | Bone drill, electrocautery, scalpel | 2.9 _ 105 RBCs or 0.87 mg of Hb is inhaled during a  typical orthopedic procedure. 60% RBCs were found  to be associated with particles >10 mm in size. Only 8% with particle <0.5 mm in size. Less than 135  lymphocytes inhaled per surgery | Clear evidence | Clear evidence | Viral |
| Bentley CD et al., 1994 [7] | Clinical human pilot study | Plate culture | Cavity preparation  Scaling procedure | High-speed handpiece | This study demonstrates that contamination from splatter and aerosol dissemination remains a  Significant hazard to dental  Personnel when high-speed  Dental equipment is used | Weak evidence | Unclear | Bacterial |
| Legnani P et al,1994 [89] | Human study | Plate method | Dental procedures | Ultrasound scaler and bicarbonate cleaner | Initial environmental conditions were mediocre which became very bad during dental procedures | Clear evidence of aerosol generation | Unclear | Bacterial |
| Heinsohn P et al 1993 [90] | Human study | Hb concentration | Common procedures in orthopedics, urology, CTVS and obstetrics | N/M | Mucous membrane of URT are likely to be exposed to aerosolized blood in the operating room | Clear evidence of aerosol generation | Clear evidence of transmission risk | N/A |
| Jewett DL. Et al 1992 [91] | Animal study (bovine) | Particle size and Hb concentration | Bone cutting and tendon cutting and coagulation | Oscillating bone saw, Hall drill, Shea drill and electrocautery | Most of the particle size were in the respirable range | Clear evidence of aerosol generation | Clear evidence of transmission risk | Viral |
| Earnest R and Loesche W, 1991 [92] | Clinical human case series | MM 10 sucrose agar plates | Cavity preparation | High speed rotary instruments | Dental aerosols produced during caries excavation contain high proportions of mutans streptococci and S. sanguis | Weak evidence | Unclear | Bacterial |
| Heinsohn P et al,1991 [93] | Animal study (bovine) | Particle size and Hb concentration | Bone cutting and tendon cutting and coagulation | Oscillating bone saw, Hall drill, Shea drill and electrocautery | Most of the particle size were in the respirable range and Hb was detected in all samples | Clear evidence of aerosol generation | unclear | N/M |
| Johnson et al.,1991 [94] | Laboratory  based | Particle concentration, particle  size, hemoglobin content in  aerosol. | Bone cutting | electrocautery, bone saw, irrigator and router | Aerosols generated by bone saw and router showed  positive HIV cultures | Clear evidence | Clear evidence | Viral |
| Micik RE et al,1969 [95] | Cross sectional | Bacterial count on agar plates | Oral examination, scaling, Tooth preparation | Hand scaler, prophylaxis handpiece, air turbine handpiece | Some dental procedures generated aerosls with bacterial concentrations that exceeded thiose produced during coughing or sneezing | Clear evidence of aerosol generation | Unclear | Bacterial |
